# Cytoskeletal prestress homeostasis is a biological principle that governs living cell structure and function

**DOI:** 10.1016/j.mbm.2026.100179

**Published:** 2026-03-03

**Authors:** Fazlur Rashid, Ning Wang

**Affiliations:** a Institute for Mechanobiology, Northeastern University, Boston, MA, USA; b Department of Bioengineering, College of Engineering, Northeastern University, Boston, MA, USA; c Department of Mechanical Science and Engineering, The Grainger College of Engineering, University of Illinois at Urbana-Champaign, Urbana, IL, USA; d Department of Communication Sciences and Disorders & Department of Pharmaceutical Sciences, Bouvé College of Health Sciences, Northeastern University, Boston, MA, USA

**Keywords:** Cytoskeletal prestress, Biological principles, Cell structure, Stiffness, Living cells

## Abstract

Over the last few decades, physical principles have been proposed to explain some biological processes and functions. However, biological principles remain elusive. A biological principle is a governing rule that guides the structure and functions of cells. Biological principles are built upon the laws of physics and chemistry, but they go beyond these laws and are unique to living matter. Here, we discuss what differentiates a biological principle from a physical principle and discuss candidates for biological principles. We review evidence from literature that regulation of cytoskeletal prestress (endogenous cytoskeletal pre-existing tensile stress) is essential for governing biological structures and functions of living cells. We propose that, in addition to the biological principles of Central Dogma and metabolism, cytoskeletal prestress homeostasis is a biological principle of a living cell across all domains of life. We propose that living cells regulate their stress and modulus to limit maximum strain on the cells. Homeostasis of endogenous energy-dependent, stress-supported systems that use cytoskeletal (CSK) prestress (the force of life) to stabilize structure represents a biological principle of a living cell that is not observed in inorganic systems, whereas other basic principles (e.g., self-assembly) are required for living systems but are also found in simpler nonliving systems. Leveraging biological principles of cells may have far-reaching implications in understanding the essence of cell life and designing effective interventions for therapeutics to advance medicine and enhance human health.

## Highlights

• This review presents evidence that cytoskeletal prestress is critical for the structure and functions of living cells.
• This review proposes that cytoskeletal prestress homeostasis is a biological principle governing cell structure and function.
• Biological principles that are built upon but go beyond physical and chemical principles are the keys to advancing human health.

## Graphical abstract

**Figure:**
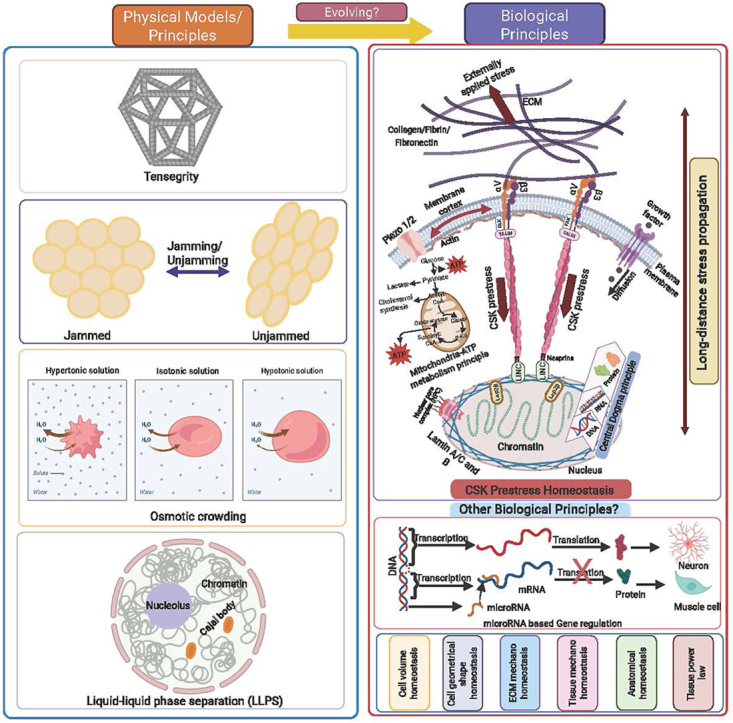

## 1 Introduction

A living cell is the basic unit of life. Hence, understanding biological principles of cells—fundamental rules governing their functions and structure—is essential. Although discoveries and findings over the last seven decades have substantially enriched our understanding of various building blocks and components of the living cell that are necessary for synthesizing proteins from DNA and RNA via transcription and translation (the Central Dogma), what are the other fundamental biological principles that guide the structure, shape, function, and behaviors of a living cell remain unknown. These components are used to form the structure and shape of the cell, sense and respond to various mechanical, chemical, and electrical signals, and execute the functions of a cell (e.g., proliferation, differentiation, and invasion). Central Dogma is a fundamental biological principle of living matter. In contrast to vertical gene transfer, which transmits genetic material from parent to offspring, horizontal gene transfer (HGT) is the lateral movement of genetic information between genomes and is important in eukaryotic genome evolution.^1^ HGT can occur among species of vertebrates,^2^ which may play a role in the evolution of organisms over millions of generations, but the rules that govern the extent and efficiency of HGT are not clear. Here, we limit our consideration of biological principles to individual cells or groups of cells in an organism within one generation, not the principle of evolution emerging naturally from variation, heredity, and differential reproduction, spanning many generations of living cells and organisms.

Living cells must obey physical and chemical principles as well as biological principles. However, biological principles of living cells remain elusive. Here, we provide a perspective that the homeostasis of cytoskeletal prestress is a biological principle of bacteria, single-celled protists, plant cells, and animal cells. Understanding the biological principles of living cells could provide insights into how cells match their structure to function and maintain resiliency.

## 2 Fundamental principles of non-living systems

### 2.1 Self-assembly

**Fig. 1:**
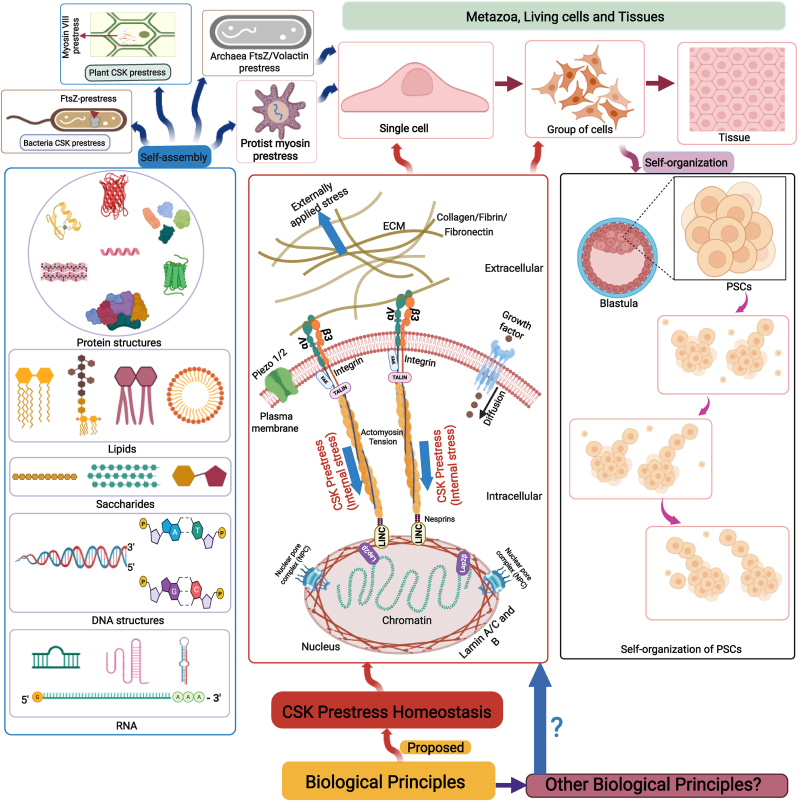
**CSK prestress homeostasis is proposed as a biological principle of living cells.** **Top left**, prestress of a plant cell, a bacterium, an archaea, or a protist. **Top right**, metazoa, animal cells and tissues. **Bottom left**, self-assembly of major biological molecules. **Bottom right**, self-organization of PSCs. **Bottom middle**, a proposed principle of CSK prestress homeostasis. ECM, extracellular matrix. PSCs, pluripotent stem cells. CSK, cytoskeleton. FAK, focal adhesion kinase. For brevity, many other focal adhesion proteins are not drawn. LAP2β, lamina-associated polypeptide 2β. FtsZ, a tubulin-like protein in bacteria. Volactin, actin-like protein.

Self-assembly is the ability to spontaneously form ordered structures in specific patterns or arrangements from interactions among individual components without external interventions. It is a process that works at equilibrium and creates static and low-energy structures.^3^ Biological molecules form reversible non-covalent bonds during self-assembly. Self-assembled structures are stable once they are formed; they may or may not require initial energy to start the process.^3^ Proteins, lipids, saccharides, DNA, and RNA readily self-assemble into complex structures 4, 5, 6, 7 (left panel of Fig. 1). It is believed that the self-assembly of these biomolecules constitutes a design principle for new materials, devices, and machines that could have significant applications in biology, medicine, and health. However, soap bubbles and other colloidal nanocrystals also readily self-assemble into complex structures.^8^^,^^9^ It is well known that these self-assembled structures are non-living because they are not made of biological molecules, nor can they reproduce. Therefore, it might be difficult to call these self-assembly processes fundamental biological principles.

### 2.2 Self-organization

Self-organization is a process in which order and pattern emerge from numerous interactions among the lower-level components of the system that is far from equilibrium and generates dynamic and continuous dissipation of energy.^10^ Self-organization is referred to as dynamic self-assembly by Whitesides.^3^ However, some researchers propose that self-organization in cellular architecture is a general principle in cellular organization.^11^ While self-assembly requires low energy, self-organization dissipates energy continuously and requires a continuous supply of energy.^12^ Self-organization has been observed both in biological systems and nonbiological systems. For example, spontaneous kinematic self-replication has been observed from pluripotent stem cells that are removed from early-stage frog blastula without using genetic manipulation^13^ (right panel of Fig. 1). While viewing self-organization as a biological principle appears logical and attractive, self-organized patterns in physical and chemical systems have also been observed in non-living systems such as the Belousov-Zhabotinsky reaction.^14^ A study demonstrates that in purified droplets of cross-linked biopolymer filaments, the inherent anisotropy drives active motor self-organizations, drop formation, and division into two droplets, suggesting that physical principles underlie self-organization in complex biological assemblies.^15^ Therefore, it is an open question whether self-organization in living cells is a fundamental biological principle.

## 3 A new proposal for biological principles of living cells

A principle should satisfy the criteria of universality, mechanism-independence, explanatory primacy, predictive necessity, and falsifiability by counterexamples. Biological principles tend to operate at the level of principles that have evolutionary history and contingency.

### 3.1 Homeostasis

Homeostasis is the ability to maintain a dynamic, stable internal environment when faced with changes in the external environment. There are plenty of examples of homeostasis in living systems: for instance, pH (i.e., hydrogen ion concentration) homeostasis, calcium homeostasis, and other ions (e.g., sodium, potassium, chloride, iron, copper, zinc, magnesium, manganese, bicarbonate, dihydrogen phosphate, hydrogen phosphate, etc.) concentration homeostasis. The requirement for Pauling's electroneutrality (a chemical principle) requires that the number of total positively charged molecules in the cell be approximately equal to that of total negatively charged molecules inside the cell, establishing homeostasis for almost all ion concentrations. However, in addition to homeostasis of pH and calcium concentration, regulation of sodium and potassium ion concentrations after an action potential^16^ has been known to be critical for normal cell function and might be more fundamental than regulation of other charged molecules. Another example is that integrin α5β1 conformational states and intrinsic affinities are differentially regulated by Mg^2+^ and Mn^2+^, suggesting that Mg^2+^ can be replaced by Mn^2+^ for strong integrin activation.^17^ Homeostasis of various molecules, like proteins, is controlled and regulated by the demands on the vital functions of the cell through establishing stability of dynamic structures.

Because a living cell must obey the laws of physics (e.g., Newton's laws and the law of energy conservation) and chemical laws of mass conservation and stoichiometry, the biological principle(s) of a living cell must be built upon these physical and chemical laws but go beyond these laws and are unique and specific for living matter. Therefore, not all processes of homeostasis are foundational: some processes of homeostasis are more fundamental than others. For example, temperature is regulated only in some animals: for endothermic animals such as birds and mammals, temperature is regulated within a certain range, maintaining its homeostasis. For the structure of the cell to meet its functional demands, the cell may follow fundamental biological principles to satisfy its structure-function relationship under external stimuli and perturbations and/or internal active processes.

Cytoskeleton (CSK) is a stress-bearing structure for living cells. For example, one such fundamental functional demand is the maximum strain and the maximum strain rate that the cell and its CSK substructures can tolerate without injury or damage. Exposed to a large strain and/or a high strain rate, the cell responds and changes its endogenous biochemical activities and gene expression through the process of mechanotransduction. The cell also likely regulates its endogenous CSK prestress (through prestress homeostasis) and/or its local modulus (through remodeling its structure) to lower the total strain. If the total strain exceeds the maximum tolerable strain, accumulation of strain-induced structural changes could lead to failures of the plasma membrane, the cytoplasm, the nuclear envelope, and the chromatin, which could lead to DNA damage, cell damage/injury, and even cell death.

### 3.2 A new perspective on CSK prestress

In this report, we propose a new perspective that the homeostasis of CSK prestress (defined as the regulation of endogenous CSK pre-existing tensile stress within a physiological range) is a fundamental biological principle of living cells, ranging from bacteria FtsZ to myosin VIII or XI in plant cells, to single-celled protists, and to animal cells (top panel of Fig. 1). We propose that CSK prestress homeostasis under physiological conditions satisfies the criteria as a biological principle, because it appears to hold across all (animal) cell types, independent of specific molecular mechanisms (e.g., myosin, actin/spectrin, intermediate filaments, microtubules, etc.), and predicts what such a system must do. Also, a genuine, sustained deviation from CSK prestress homeostasis without restoration would falsify it.

Before we present experimental evidence for CSK prestress regulation of living cells, we would like to discuss the distinction between "tension" and CSK prestress. "Tension" has different meanings to different researchers. "Tension" may be separated into plasma membrane tension and cortex tension.^18^ Membrane tension is a useful parameter, especially when one studies physical mechanisms driving plasma membrane activities and functions. Some researchers use the term "CSK tension" to represent CSK tensile stress; with this definition, CSK tension has the unit of stress (force per unit area). For this reason, we propose that "CSK tension" is equivalent to CSK stress, not the tension (force per unit length) that is derived from surface tension or plasma membrane tension.

The experimental evidence for "tensional homeostasis" was first published in the 1990s for a group of fibroblasts cultured in 3D collagen gels^19^ and later in single cells and a cluster of cells on 2D surfaces.^20^ In these papers, the term "tension" was loosely referred to as "force"^19^ or "contractile moment".^20^ The term "CSK tension" was loosely referred to as tractions in a report in which a positive feedback loop is established that switches on the malignant phenotype in mammary epithelial cells.^21^ A recent review proposes that the ability of living organisms to keep tension at certain specified values is tensional homeostasis and that deviation from this causes diseases at different structural levels.^22^ This tensional homeostasis ranges from myosin II contractile force at the molecular level to higher levels in living organisms.^23^

It is noteworthy that membrane tension and CSK prestress are two different variables. Membrane tension (the sum of plasma membrane tension and cortex tension) may be regarded as a proxy index of the CSK prestress in the circumferential direction in the thin membrane cortex. The thin membrane cortex consists of the F-actin-myosin network that forms a very thin layer (tens of nm to ∼200 nm) of the cytoskeleton and contributes to the stiffness of the cortex.^18^ CSK prestress includes normal and shear stresses that are in the membrane cortex CSK and in the deep cytoskeletal filaments that are connected to the nuclear envelope via the LINC (Linker of Nucleoskeleton and Cytoskeleton Complex) to help stress propagation/transmission from the cell surface into the nucleus to the chromatin and nucleoplasm. It is noteworthy that the prestress in the deep CSK (but not the prestress in the cortex) is critical for long-distance stress propagation from the cell surface into the nucleus for direct mechanotransduction in the nucleus.

It is possible to estimate the magnitude of CSK prestress in a living cell(s) on 2D surfaces by quantifying tractions and cell spreading areas 24, 25, 26 or in 3D cultures using various traction force probes.^27^ It is important to note that cytoskeletal prestress and thus tractions are tensorial and highly heterogeneous, which can only be described in 2D or 3D prestress and traction maps. However, tractions are often plotted as a scalar “RMS (root mean square) Traction” to represent the contractile state of the cell. In this review, we use the word prestress as a generic term to represent the contractility of the cell. We also use the word “force” as a generic term for “mechanical loading”.

#### 3.2.1 Bacteria and archaea CSK prestress

Although myosin II-mediated CSK prestress is absent in bacteria and archaea, prestress via other mechanisms might still be at play. The cytoskeleton of bacteria includes (but is not limited to) tubulin-like protein FtsZ, actin-like protein MreB,^28^ and other actin-like proteins.^29^ However, the spatial localization of cytoskeletal components in bacteria appears to be different from that in eukaryotes.^30^ For example, bacteria have flagellar motors that generate rotatory motions to propel the movement of the bacteria,^31^ but they do not have myosin or kinesin/dynein-like motors that generate stepwise linear motions. Instead, bacteria are proposed to generate FtsZ-ring force to exert CSK prestress to overcome turgor pressure and rigid cell wall during division.^32^ However, another model suggests that the FtsZ-ring force only indirectly drives bacterial division by regulating cell wall metabolism.^33^

Archaea that have both FtsZ CSK and volactin CSK^34^^,^^35^ are proposed to be the life form that transitioned from FtsZ-based CSK prestress in bacteria to actin-based CSK prestress in eukaryotes. Recent evidence suggests that archaea use both mechanisms to develop tissue-like multicellular structures under mechanical compression.^36^ (top left panel of Fig. 1).

#### 3.2.2 Plant CSK prestress

Like animal cells, plant cells have microtubules and actin filaments as well as myosin (myosin XI and myosin VIII, as shown in the top left panel of Fig. 1) and kinesins. Myosin XI motors in plants, like myosin V in animal cells, function to drive transport and cytoplasmic streaming that is critical for plant cell growth.^37^ Genetic analysis in Arabidopsis has firmly established that myosin XI-driven movements are necessary for cell growth, polarity, organelle distribution and shape, and actin organization and dynamics.^38^^,^^39^ Myosin VIII, on the other hand, functions as a tension generator (i.e., prestress) at the cell cortex.^40^ Interestingly, when plant cells lacking walls are confined in an elongated geometry, actin organization in those protoplasts depends on the organization of the microtubule network but not vice versa.^41^ Recent advances suggest that actin filaments and microtubules, together with their associated proteins, and the CSK prestress play a critical role in the homeostasis of 'stress' (i.e., hyper-salinity, dehydration, osmotic stress, or high/cold temperature) response of plant cells.^42^

#### 3.2.3 CSK prestress in single-celled protists

**Fig. 2:**
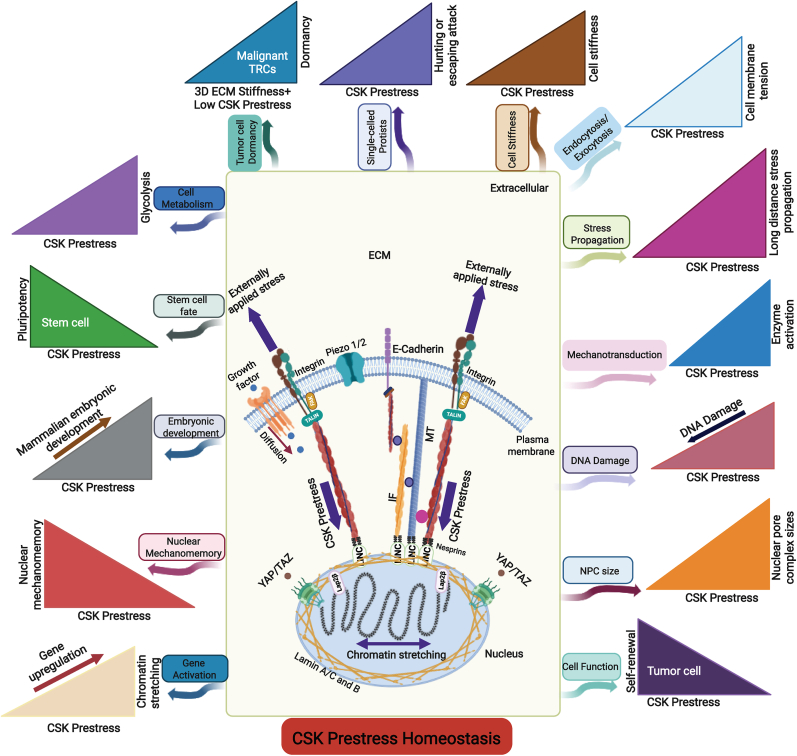
**Cell functions are regulated by CSK prestress homeostasis.** Experimental evidence shows that cell structure and functions are regulated by CSK prestress. TRCs, stem cell-like malignant tumor-repopulating cells. YAP/TAZ, Yes-associated protein/transcriptional coactivator with PDZ-binding motif. MT, microtubule. IF, intermediate filament. LINC, Linker of Nucleoskeleton and Cytoskeleton. Membrane tension is a proxy index of CSK prestress and is proportional to the CSK prestress in the circumferential direction in the membrane cortex.

Myosin-dependent CSK prestress in protists like amoeba is regulated during cytokinesis^43^ and migration.^44^ Studies on the single-celled, ciliated protist *Lacrymaria olor* reveal that the cell hunts for prey by rapidly extending its long neck via a layered cortical microtubule cytoskeleton strategy^45^ and the actomyosin-mediated CSK prestress,^46^ as shown in Fig. 2. Another study on the single-celled, ciliated organism *Stentor roeselii* demonstrates that the cell rapidly escapes a physical attack of microparticles by bending its body, altering cilia beating, contracting its body, or detaching from its anchorage.^47^ The adoption of different responses (bending, beating, contracting, or detaching) to the same mechanical challenge for this organism to return to its original unperturbed homeostasis state represents a primitive behavior of mechano-intelligence, reminiscent of the intelligence defined by William James. These studies suggest that the regulation of the actomyosin-mediated CSK prestress is essential for the life and death of these primitive protists.

#### 3.2.4 CSK prestress regulates cell stiffness

Over the last few decades, accumulating experimental evidence from many labs demonstrates that CSK prestress in animal cells regulates cell stiffness ^24^^,^^25^^,^^48^, ^49^, ^50^, ^51^. When CSK prestress is quickly elevated, cell stiffness is increased; when CSK prestress is quickly lowered, cell stiffness is decreased (Fig. 2). The CSK, under a local external load, reorganizes and rearranges its structural elements (i.e., CSK filaments) relative to each other until a new mechanical equilibrium is reached. The higher the prestress, the more stable the whole structure, the higher the resistance to external loading, and hence the higher the stiffness, and vice versa.

At the subcellular and molecular levels, the linking proteins such as plectin that links microtubules to intermediate filaments,^52^ ACF7 that links F-actin with microtubules,^53^ Microtubule-associated proteins (MAPs) such as MAP1, 2, 4 family and Tau proteins that crosslink microtubules with actin filaments,^54^ α-actinin that crosslinks F-actins,^55^ all contribute to the stress transmission from actomyosin to other filament systems and the stiffness of the cell. The detailed role of single myosin II protein activation in actin CSK stiffening is not clear but it may be related to catch bonds^56^ in the actin filament and/or actomyosin binding site, where the number of noncovalent bonds is increased by the myosin II force. The regulation of cell stiffness or modulus by CSK prestress is also experimentally demonstrated by many labs in many cell types under various conditions 57, 58, 59, 60, 61, 62. It is well known that CSK prestress in animal cells is downstream from key processes of actin polymerization and myosin II contractility, which in turn, is regulated by processes of activation of myosin light chain kinases or phosphatases, and Rho kinases, etc.

#### 3.2.5 Long-distance stress propagation enabled by CSK prestress

When two proteins bind to each other, the total number of noncovalent bonds determines the specificity of the binding and the stability of the protein-protein complex.^63^ This observation can be extended to DNA and protein or DNA and RNA. The distances between these noncovalent bonds are extremely short and are ∼0.2 nm. At a longer length scale of micrometers, membrane tension does not mediate long-range intracellular signaling, as the tension decays rapidly within ∼1 μm from the local load perturbation.^64^ This short-range stress impact is consistent with St. Venant's principle of stress decaying with the inverse of the square of the distance.

However, when a local stress of physiological magnitude is applied to the focal adhesion of the cell, due to the presence of the CSK prestress, the applied stress decays much more slowly with distance (∼with the inverse of the distance). The applied stress propagates to tens of micrometers, from one side of the nucleus to the other side of the nucleus, to exert its impact on most parts of the cell 65, 66, 67, 68. The long-distance force propagation finding has been quantitatively confirmed by theoretical and modeling analysis.^69^^,^^70^ Modulating the CSK prestress of the cell regulates the distance of the stress propagation (Fig. 2), suggesting that CSK prestress homeostasis is critical in the unique phenomenon of long-distance stress propagation in the cell.

#### 3.2.6 Rapid cellular mechanotransduction in the cytoplasm depends on CSK prestress

There is rapid cellular mechanotransduction in the cytoplasm (∼100-300 ms after a stress application) in activating enzymes Src and Rac that depends on long-distance stress propagation.^71^^,^^72^ This mechanical stress-induced rapid enzyme activation in the cytoplasm is ∼100-fold faster than the growth factor epidermal growth factor (EGF) or platelet-derived growth factor (PDGF) induced Src or Rac activation and depends on the level of the CSK prestress.^71^^,^^72^ Increasing CSK prestress increases enzyme activation, shown in Fig. 2. In addition, a recent report finds that actomyosin contractions (i.e., the CSK prestress) in the actin cortex induce rapid global membrane tension propagation and local pulling forces on cell membranes alone only induce local deformation,^73^ consistent with the result that CSK prestress regulates long-distance rapid Rac activation on the cell membrane.^72^

#### 3.2.7 Chromatin-deformation-dependent gene regulation depends on CSK prestress

Numerous studies have shown the existence of indirect mechanotransduction pathways from the cell surface to the nucleus involving membrane ion channels such as TRPV4, Piezo1/2, focal adhesion molecules, and relay molecule YAP/TAZ (Yes-associated protein/transcriptional coactivator with PDZ-binding motif) 74, 75, 76, 77. However, a direct force transmission pathway from focal adhesions to chromatin via the CSK and the LINC has been demonstrated in living cells ^70^^,^^78^, ^79^, ^80^.

Importantly, the rapid gene transcription induced by short durations (∼minutes) of stress of physiological magnitudes applied via the integrins depends on the direction of the applied stress^70^^,^^78^ and the CSK prestress,^70^ but does not depend on calcium ion channels or YAP/TAZ.^79^^,^^80^ Further experimental evidence shows that how much a gene is rapidly upregulated depends on the extent of the stretching (varying from 2% to 12% tensile strains) of the chromatin domain. In contrast, compressive strains on the chromatin domains downregulate gene transcription via condensing the chromatin^80^. In addition, intranuclear protein LAP2β that connects the nuclear lamins with the chromatin is shown to be a mechanosensor that transmits the stress from the nuclear envelope to the chromatin to deform the chromatin ^80^ (middle panel of Fig. 2).

It is known that large strains and high strain rates (e.g., a 50% compressive strain that is applied instantaneously) might cause DNA damage,^81^ and inhibition of actomyosin contractile prestress can minimize DNA damage^82^ (Fig. 2). Cell shape/nuclear shape deformation sensing is regulated by cPLA2-dependent cell contractility in confined cell migration 83, 84, 85.

#### 3.2.8 Nuclear mechanomemory after load release is regulated by CSK prestress

Initially the idea of mechanomemory was proposed as the mechano-sensed information that permanently changes the phenotype of the cells via epigenetic changes in the nucleus.^86^ However, this notion can be extended as mechanomemory is a sustained response (much longer than the time constant of material viscoelasticity) of the cell, the tissue, the organ, or the organism that continues after force cessation. Experimental findings suggest that the duration of mechanomemory can vary from minutes to hours, to days, to months, and to years (e.g., fibrosis), depending on the duration of the mechanical stimulus^87^ and the types of biological entities (e.g., cells vs extracellular matrices in the tissue).

An early report shows that stem cells exhibit mechanical memory, and their transcription factors remain activated as if the cells were still on the stiff substrate when they are switched from a stiff substrate to a soft substrate.^88^ Similarly, after the cells are switched from stiff to soft substrates, mesenchymal stem cells exhibit fibrotic mechanomemory via myocardin-related transcription factor-A-dependent elevation of microRNA (miR-21).^89^ However, it is known that gene transcription continues for tens of minutes after cessation of the applied stress that is applied for only a short period of 2 min or 10 min^87^_,_ such that there is no elevation of YAP/TAZ translocation into the nucleus.^80^

It turns out that stretching chromatin by applying stress via integrins for a few minutes can increase chromatin and nucleoplasmic protein mobility inside the nucleus, leading to nuclear mechanomemory (Fig. 2) tens of minutes after cessation of stress.^87^ A recent report finds that viscoelastic substrates improve reprogramming efficiency via enhancing epigenetic remodeling and cellular mechanomemory.^90^ Furthermore, it is shown that nuclear pore complexes (NPCs) facilitate duration of the mechanomemory in chromatin and nucleoplasm protein diffusivity,^87^ nucleocytoplasmic transport by perforating the nuclear envelope, and fast volume change during osmotic pressure challenge causes directional fluid flow through NPCs.^91^^,^^92^

This finding is supported by mechanomemory data from a method using a direct magnetic nanoparticle force probe that is microinjected into the nucleus to bind specifically to the chromatin^93^. Mechanomemory of RNA Pol II diffusivity and activity persists for 30 min after stress cessation,^93^ explaining the persistent gene upregulation 30 min after stress release.^80^ The duration of nuclear mechanomemory and the net change in stress-induced YAP translocation decrease with substrate stiffness,^93^^,^^94^ likely because nuclear pore complex sizes are increased by the elevated CSK prestress on the stiff substrate (Fig. 2).^95^^,^^96^

#### 3.2.9 Cell functions depend on CSK prestress

Migrating amoeba are known to generate tractions, and both filamentous cortical layer and large fibrils of the actomyosin network within the cells contribute to the tractional stress generation.^97^ In addition, CSK tractional stress is higher in stationary cells than in migrating animal cells on planar surfaces.^98^ Interestingly, there is evidence that the CSK prestress in intercalating cells in Drosophila regulates myosin II dynamics because myosin II is recruited and stabilized at the region of high CSK prestress.^99^ Cancer cells generate 3D tractions (via the CSK contractile prestress) to activate protease-dependent invasion.^100^ The CSK prestress also controls cell cycle progression in endothelial cells.^101^ A published report shows that myosin II-mediated CSK contractile stresses sustain and polarize hematopoiesis from stem cells and progenitor cells.^102^ Several labs have independently demonstrated that the CSK prestress regulates the contractile-adhesion balance in myocytes,^103^ cell differentiation,^58^ stress-fiber-dependent focal adhesion maturation,^104^ and prestress in the nucleus via actin-nesprin2 linkage.^105^

The CSK prestress can also regulate metabolism of cells, when the cells are switched from stiff to soft substrates, a downregulation of glycolysis is observed via the disassembly of stress fibers,^106^ and thus a reduction in the CSK prestress (Fig. 2). In addition, when E-cadherin is under external tensile forces, it forms a complex with LKB1, AMPK, and vinculin; activation of vinculin strengthens the cytoskeletal anchorages and enhances the CSK contractile prestress, which in turn activates AMPK, which increases glycolysis and ATP (Adenosine Triphosphate) generation.^107^

Experimental evidence shows that stem-cell-like self-renewing tumor repopulating cells are incredibly soft and generate low CSK prestress,^108^^,^^109^ like the soft embryonic stem cells 110, 111, 112 (Fig. 2). Cell prestress is shown to drive mechanomemory of squamous cell carcinoma via distinct kinase signaling.^113^ In addition, cytoskeletal contractile prestress is critical in cytotoxic T cells killing tumor cells.^114^ Malignant tumor-repopulating cells can evade cytotoxic T cell killing through a mechanical softness mechanism by impairing perforin pore formation. Elevating tumor cell stiffness restores T cell-mediated cytolysis of tumor-repopulating cells.^115^ A recent report further demonstrates that cytotoxic T lymphocytes and T leukemic cells are resistant to perforin-mediated killing by being soft,^116^ by regulating CSK structure, and by their CSK prestress. The soft and low-prestress-generating malignant tumor cells are more metastatic in zebrafish^117^ and mouse^108^^,^^109^ models.

Interestingly, a living cell can integrate both mechanical signals and growth-factor-induced signals via the action of CSK prestress.^71^ The integration of mechanical and biochemical signals is critical in the direct transdifferentiation from human fibroblasts to heart valvular endothelial cells for engineering human-cell-seeded pig aortic valves.^118^ Human mesenchymal stem cells respond to matrix stiffening by increasing nuclear tension, causing histone-modification-based epigenetic remodeling.^119^ During early mammalian embryonic development, CSK contractile prestress is increased in human and mouse embryos during compaction.^120^

All the above discussion on the CSK prestress does not suggest that it is a static variable. In fact, it is very dynamic and conserved across species: fluctuations and oscillations in the CSK prestress are observed in amoeba,^97^ in mouse embryonic fibroblasts,^121^ and in early developing zebrafish and mouse embryos.^122^ Together, these findings suggest that cell migration, invasion, proliferation, differentiation, metabolism, integration of mechanical and biochemical signals, nuclear epigenetic remodeling, and early embryonic development are all regulated by the CSK contractile prestress.

## 4 Emerging biological and physical principles

### 4.1 Is a biological principle distinct from a physical principle?

**Fig. 3:**
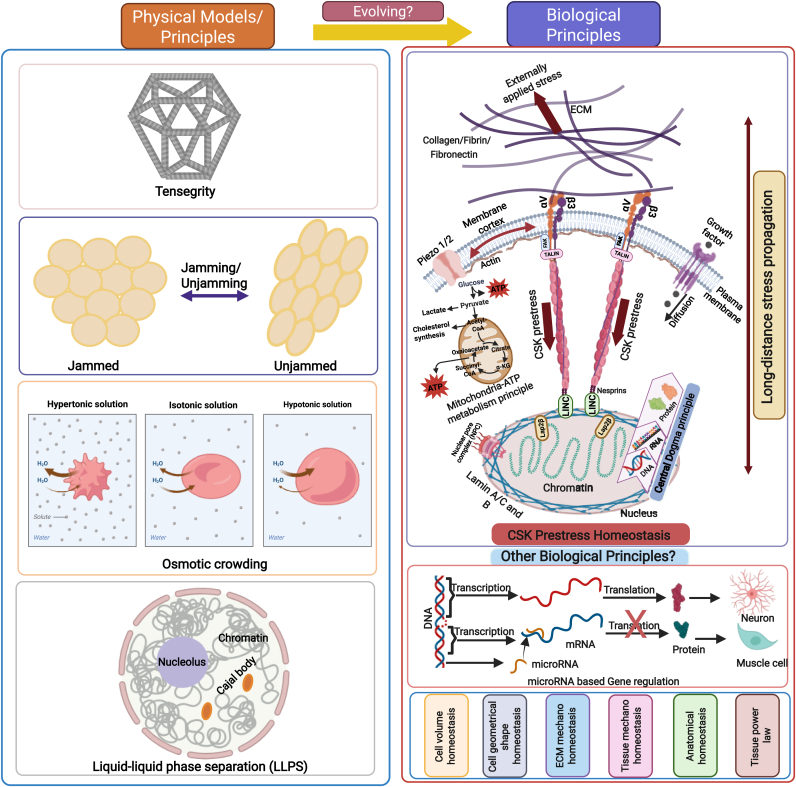
**Comparison of biological principles with physical principles.** **Left**, physical models/principles. **Top right**, CSK prestress homeostasis principle. **Bottom right**, other candidate principles of cells and tissues. Together with the Central Dogma principle, the Metabolism principle, the biological principle of CSK prestress homeostasis, and other candidate biological principles may help better design interventions for therapeutics to advance medicine and health.

Over the last several decades, several physical principles for living cells have been proposed that are supported by experimental results: tensegrity,^123^ soft glass rheology,^124^ jamming/unjamming transition,^125^ liquid-liquid phase separation,^126^ osmotic crowding^127^ (Left panel of Fig. 3). While these physical models or principles are extremely valuable in explaining certain emergent behaviors and functions of living cells and in understanding the physical rules that the living cells must follow and obey, numerous non-living structures or materials use these principles. In other words, the fundamental "living" requirement is missing from these physical principles and models.

The fundamental "living" requirement is likely to include an assemblage of these essential features: the process of DNA to RNA to protein (right panel of Fig. 3), Metabolism (right panel of Fig. 3), Movement/contractility by endogenous forces, Self-organization, External signal sensing, Volume regulation, Geometrical shape regulation, Endocytosis/exocytosis (eukaryotic cell), Autophagy (eukaryotic cell), Response to stimuli, Regulation of internal milieu (homeostasis), Replication, Reproduction, Development, Regeneration, Self-repair, Memory, Gene Regulation, Differentiation (multi-cellular eukaryotic cells), and Evolutionary adaptation.

The proposition of myosin II-mediated CSK prestress homeostasis is built on the conditions that myosin II contractile activity is driven by ATP, and that homeostasis implies that biological feedback loops must exist to maintain a stable and controlled internal state of the CSK prestress. Both are essential features of a living (eukaryotic) cell. It is of note that force homeostasis is related to but different from the concept of force balance because force homeostasis refers to a state in which the force is regulated within a range. Homeostasis is also different from equilibrium, as all living cells are far from equilibrium.

As we have stated, any biological principle must obey the laws of physics and chemistry and physical principles. In this regard, the principle of the CSK prestress homeostasis proposed here is built on and extended from the cellular tensegrity model.^123^ For instance, the simple six-strut tensegrity model^128^ alone does not predict the long-distance stress propagation behavior of the cell, which only exists in a heterogeneous complex material in the cytoplasm that includes long and stiff prestressed stress fibers and soft CSK networks 69, 70, 71, 72. Furthermore, the requirement for the supply of ATP for activation of myosin II and the feedback loops in the cytoplasm to maintain the homeostasis of the CSK prestress are not intrinsic to the tensegrity model.

It is important to note that cell volume regulation is critical for cell structural integrity and function. The "pump-leak model" (PLM) shows volume regulation in the presence of osmotic challenges.^129^^,^^130^ This classical PLM is extended by a mechanosensitive PLM that discovers that a mechano-osmotic coupling by a membrane tension homeostasis mechanism leads to cell volume fluctuations in response to fast cell shape changes.^131^ Cell shape changes, critical for many cell functions, are driven by membrane tension that induces exocytosis^132^ and adhesion positioning during cell spreading,^133^ promotes collective cell migration,^134^ and is critical for mechanotransduction.^135^ Cell volume regulation is conserved across all domains of life, and cyclic di-AMP is proposed as a master regulator of cell volume in bacteria.^136^ We postulate that cell volume homeostasis is a candidate for a fundamental biological principle of a living cell (Fig. 3).

### 4.2 Is metabolism a biological principle?

Because metabolism is the fire of life^137^ for all living matter, essential for making new substances such as ATP, it is logical to view metabolism, that is tightly regulated within an individual living cell, as a biological principle. However, some researchers argue that metabolism is more accurately described as an ensemble of ordered and organized biochemical reactions, not as a biological principle. To address these two distinct views, one might first ask a different question: Is there any non-living matter that uses metabolism for its primary energy source? Take viruses as an example. Viruses, although they undergo evolutionary adaptation in a host cell, lack metabolism, and they hijack the host's (the living matter) metabolism entirely. Autocatalytic chemical networks are spontaneous chemistry, not metabolism. Artificial protocells or synthetic biology constructs in sophisticated cell-free systems or lipid vesicles with reconstituted enzymes still must use components extracted from living systems and cannot create de novo non-living metabolism. Therefore, nonliving matter does not use metabolism that requires the integrated, evolved, self-maintaining organization found only in living systems. In contrast, mitochondria and chloroplasts are evolutionary remnants of once-living bacteria, now obligately dependent on their host cells but still retaining metabolic machinery inherited from their free-living ancestors; hence, they are not counterexamples but are degenerate living systems. Because metabolism meets the criteria of a principle and only exists in a living cell, we propose that metabolism is a fundamental biological principle. Because metabolism provides the chemical energy for actomyosin force generation, it is responsible for the generation of CSK prestress. However, metabolism per se does not guide the cell for its structure, shape, functions, and behaviors, such as directed movement (migration and invasion) in animal cells. There is evidence that metabolism is regulated by the CSK prestress^106^ and that metabolism and the CSK prestress are mutually dependent on each other.^138^

### 4.3 Prestress homeostasis

CSK prestress is regulated within a physiological range to maintain homeostasis. How a living cell sets its prestress range is of interest. A living cell is assumed to set the range of its prestress to meet the demands for regulating essential features of the cell by altering its actomyosin contractility and distributing the contractile forces along its cytoskeletal filament systems (e.g., stress fibers; prestressed actin bundles, membrane actin cortex, and intermediate filament systems) and the nuclear lamins and the chromatins. The prestress is balanced by microtubules and cortex stiffness inside the cell, and by the extracellular matrix (ECM) outside the cell. For multiple cells, the prestress is also balanced by neighboring cells that come into contact.

In an adherent and spread human airway smooth muscle cell, the baseline pre-set magnitude of CSK prestress is ∼1000 Pa, which can be regulated to several-fold lower (∼300 Pa) and one-fold higher, 2000 Pa, by relaxing or contractile agonists without changing the cell spreading area,^50^ balanced by the microtubules and the ECM.^26^ This range of prestress is necessary for performing the physiological functions of regulating airway caliber during coughing to rid foreign irritants. However, it is well-known that smooth muscle cells (that line various types of lumens in the body) have a baseline tone (a sustained rhythmic oscillatory contraction (and prestress) of small amplitudes)^139^ and that airway smooth muscle cell tone is critical for airway responsiveness in both healthy mice and humans.^140^ The set range for CSK prestress could also vary with the differentiation of the cell for multicellular eukaryotic cells. It is important to note that homeostasis is a regulated response such that the system returns to appropriate set ranges after the perturbation. The set ranges are not necessarily narrow. For example, human arterial blood pressure increases by more than 2-folds during exercise but returns to the normal range after exercise.

Malignant stem cell-like tumor cells in spheroids exhibit low CSK prestress.^108^^,^^109^ When malignant tumor cells are being treated with an anti-cancer synthetic retinoid, the 3D CSK prestress goes to zero even before the cells' apoptosis can be measured.^141^ In contrast, if the myosin II-mediated CSK contractile prestress pushes and pulls the migrating cells too hard through a very narrow constraining pore, the cells undergo apoptosis.^142^ Recent experimental evidence suggests that myosin II-mediated CSK prestress plays a key role in the mechano-evolution of heritable changes in chromosome numbers.^143^^,^^144^ This suggests that CSK prestress is fundamental for biological processes.

Other biological principles have recently been proposed. For example, microRNA^145^^,^^146^ is regarded as "a completely new principle of gene regulation that turned out to be essential for multicellular organisms, including humans"^147^ (bottom right panel of Fig. 3).

### 4.4 Is CSK prestress homeostasis distinct from principles of Central Dogma and metabolism?

In addition to satisfying the criteria that Central Dogma and metabolism meet as biological principles, CSK prestress homeostasis possesses other distinct or unique features: prestress is a tensor that captures spatial information about cell organization, not just information “flow” like Central Dogma and energy “flow” like metabolism. Prestress originates from active intracellular forces; prestress operates on timescales relevant to cellular functions (milliseconds to days), bridging molecular dynamics (microseconds) and developmental processes (hours to days). This multi-scale integration is crucial; prestress couples mechanical to biochemical signaling, because the mechanical state regulates gene expression, metabolism, etc., this bidirectional coupling makes prestress integrative. These unique features suggest that prestress could function as the third foundational pillar, the mechanical and spatial organizing principle that complements heredity and energetics. As such, we propose that CSK prestress homeostasis is one of the three fundamental biological principles: Central Dogma defines how biological information flows, metabolism specifies how biological energy flows, and CSK prestress homeostasis governs how biological space is organized, directed, changed, and maintained.

### 4.5 Is prestress homeostasis merely a derivative of deeper principles of energy dissipation, information processing, or CSK self-organization?

CSK prestress homeostasis may trace its roots to energy dissipation maintenance of order based on thermodynamics,^148^ negative feedback regulation based on control theory and information processing 149, 150, 151, and self-organization of motor-driven CSK filament networks from active matter physics ^15^^,^^152^, ^153^, ^154^, ^155^. These are the physical substrates and control theory that make prestress homeostasis possible. However, at the biological level specific to living cells, prestress homeostasis is genuinely autonomous. This is because the prestress set-range is biologically specified, because its violation corresponds to loss of cell identity and pathology, because it is multiply realized across evolution, and because it couples mechanical state to gene expression and function in ways that have no physical or information processing analog. We thus propose that CSK prestress homeostasis is a principle of living cells rather than a derivative of deeper principles.

## 5 Alternative theories/models of mechanotransduction

### 5.1 Plasma membrane mechanics model

Forces are sensed primarily at the plasma membrane through changes in membrane tension, curvature, and lipid bilayer composition. Mechanosensitive channels like Piezo1/2 and TREK/TRAAK (mechanosensitive, thermo-sensitive two-pore domain potassium ($K_{2P}$) channels are embedded in the plasma membrane and might respond to bilayer deformation directly, independent of the CSK.

### 5.2 CSK poroelastic model

Cells are treated as biphasic materials, a porous elastic solid matrix permeated by cytoplasmic fluid. Mechanical forces drive fluid redistribution through the cytoskeletal meshwork, generating pressure gradients and flow that can activate signaling. This model emphasizes the role of intracellular fluid dynamics rather than solid-state force transmission and has been particularly applied to understanding rapid mechanical responses in chondrocytes^156^ and during cell migration.^157^

### 5.3 Stochastic mechanosensing

A theoretical perspective argues that mechanosensing is not localized to any specific structure but emerges from stochastic fluctuations and collective behavior of many molecular bonds across the cell surface. Individual binding events are probabilistic and force-sensitive, and mechanosensing is a statistical property of the ensemble.^158^

### 5.4 The molecular clutch model

The molecular clutch model is a mechanotransduction framework describing how cells sense substrate stiffness through the dynamic coupling between myosin motor pulled actin CSK and ECM via integrin-based adhesions that govern cell function in response to internal and external stimuli.^159^

These alternative models of mechanotransduction are complementary to the CSK prestress homeostasis postulate and may be unique at different time and length scales.

### 5.5 When does CSK prestress become secondary?

Living cells under some conditions where CSK prestress may not be dominant: cells under acute osmotic stress where volume changes drive rapid mechanical responses, cells in hyperinflammatory responses (e.g., cytokine storm) to CAR T-cell therapy, malignant tumor cells prior to apoptosis (tractions become negligible),^141^ and cells in late-stage cirrhosis (a pathological condition). Additionally, senescent cells, which accumulate in chronically inflamed tissue through the senescence-associated secretory phenotype (SASP), show profound and permanent cytoskeletal remodeling with flattened morphology, reorganized actin, and altered nuclear mechanics that do not restore. A report demonstrates failure of the mechano-adaptive feedback loop essential for prestress homeostasis in senescent fibroblasts.^160^ A recent paper reveals that senescent bone marrow stem cells show substantially reduced intracellular prestress and impaired mechanical behavior.^161^ These senescent cells are metabolically active and viable and may have permanently exited the normal mechanical state. Taken together, these findings paint a compelling picture: senescent cells lose the ability to maintain cytoskeletal prestress within a homeostatic range. The disruption manifests either as insufficient prestress (replicative/oxidative senescence that reduced myosin II, loss of traction) or excessive/dysregulated prestress mediated CSK stiffness elevation in progeria, where RhoA is activated.^162^ In both cases, the mechano-feedback loops that would normally restore the set-range are broken. This is evidence for the postulate that prestress homeostasis is a fundamental biological principle and that its failure is a hallmark of the senescent state.

## 6 Other candidates for biological principles of cells and tissues

Other proposed biological models that are candidates for biological principles include geometrical shape homeostasis^163^ (at the cell/tissue level), ECM homeostasis,^164^ tissue mechanohomeostasis,^165^ anatomical homeostasis,^166^ and tissue power law^167^ at the tissue level (bottom right panel of Fig. 3). In the ECM or tissue mechano-homeostasis model, the stiffness of the tissue (such as the artery) is maintained within a physiological range. ECM and tissue mechano-homeostasis refer to homeostasis of tissue stiffness, which is a quantity different from tissue residual stress. Tissue residual stress was discovered when the arteries were removed from the body to reduce blood pressure and shear flow stress.^168^ Residual stress is the stress that is residual after external stress is removed. Cellular prestress, a similar concept, is actomyosin-dependent CSK stress that exists before external stress is applied. The homeostasis framework may be applied to both quantities.

Because strain is the ratio of stress over modulus, this relationship suggests that stress (external stress and endogenous CSK prestress) and modulus (stiffness) can be independently regulated by the organism, such as the human body, over different time scales to keep the maximum strain on the cell and the tissue below the structural failure magnitude.

The non-linear stress-strain relationship mediates stiffening response and undergoes structural remodeling via alterations in polymerization dynamics of individual CSK and ECM molecules, which also contribute to the modulus of the cells and the tissues. In the tissue power law model, the importance of CSK prestress-induced collagen strains in suppressing collagen degradation is demonstrated by inhibition of myosin-II mediated contractile strains, which fits the scaling of the tissue power law and reverses strain-induced suppression of collagenase activity in tissues.^167^ It is demonstrated that there is no association between collagen density in tumor tissues and poor prognosis in cancer patient survival. However, there is an association between collagen density and fibrotic gene expression.^169^ Tumor tissue stiffening responses occur due to the elevation in collagen density, but they do not promote or accelerate cancer progression. As such, it is proposed that a stiff tumor stroma, because of elevated collagen density, is a physiological fibrotic response from the human body to inhibit tumor growth and invasion into the nearby normal tissues.^170^

This interpretation is supported by the findings that stiff ECM promotes malignant tumor repopulating cell dormancy,^171^ and soft matrix promotes soft and low prestress-generating tumor cells to grow and metastasize.^172^ Numerous reports from many labs over the last two decades show that tumor cells, especially malignant stem-cell-like tumor cells, are softer than normal cells and non-invasive tumor cells 173, 174, 175, 176, 177, supporting the tumor cell softness model.^170^ A recent report reveals that high viscosity but not high elasticity circulating tumor cells favor dissemination and metastatic extravasation,^178^ in accord with but extending the tumor cell softness model. Experimental findings from numerous labs are inconsistent with the model that stiff tumor stroma tissue promotes CSK tension, cell invasion, and growth to drive cancer progression ^144^^,^^169^^,^^179^, ^180^, ^181^, ^182^, ^183^, ^184^, ^185^, ^186^. Clinical trials using the strategy of lowering tumor stroma stiffness have not produced therapeutic benefits in cancer patients.^187^^,^^188^ In contrast, using the tumor cell softness strategy to fabricate tumor-cell-derived drug-containing extracellular vesicles has produced beneficial outcomes in late-stage lung cancer patients.^189^^,^^190^ Nevertheless, more in vivo experiments and clinical interventions are needed to determine which model (the stiff tumor stroma model, the tumor cell softness model, or an alternative model) is more appropriate for describing the mechanical mechanisms of cancer progression in patients.

Because both the cells and the ECM are viscoelastic,^191^ the responses from the cells to a quick application of constant stress (i.e., a step-function stress, which would lead to a creep response) are expected to be drastically different from those responses to a quick and constant strain (i.e., a step-function compressive strain, which would lead to stress relaxation). In a similar vein, the cell's response to oscillatory stress is expected to be different from oscillatory strains. Additionally, because the cells can remodel their structures to alter their moduli, at longer durations (say, many minutes to hours and days) of the applied stress or strain, the remodeling biological processes will become more important, and they will be expected to be drastically different if the perturbation is a stress or a strain.

For the models of cell geometrical shape homeostasis and anatomical homeostasis, some aspects of them may be related to and dependent on the principles of CSK prestress homeostasis and cell/tissue modulus homeostasis. For example, it is well known that cell spreading and thus cell geometrical shape changes that control cell life and death^192^ are regulated by the CSK prestress.^193^ However, CSK prestress can also be regulated by independent cell shape changes. Therefore, cell shape and CSK prestress are mutually regulated. Cell shape homeostasis and anatomical homeostasis may depend on other yet-to-be-discovered fundamental principles.

Recently generalized biological principles on the evolution of life have been proposed.^194^^,^^195^ However, these "principles" are either too abstract to be useful for predicting or explaining specific cell behaviors and functions, or they are not fundamental biological principles. For example, development, reproduction, and regeneration that are proposed as biological principles in one report^166^ are biological processes that are driven by the underlying biological principle of CSK prestress homeostasis,^120^^,^^122^^,^^196^^,^^197^ and therefore, it is difficult to call these processes fundamental "governing principles" of cells. Biological principles (of a living cell) are the governing rules that guide the structure, shape, functions, and behaviors of the cell.

Cell membrane tension (a proxy index of the CSK prestress in the cortex along the circumferential direction) at the cell-medium interface but not at the cell-cell adhesion is elevated, which drives human embryo compaction^120^ and zebrafish and mouse embryos generate periodic 3D traction oscillations (∼15 min per oscillation) that are associated with early development,^122^ suggesting the CSK prestress homeostasis drives development. Smooth muscle cell contractility in the outer follicle drives follicle contraction during ovulation,^196^ indicating it drives reproduction. Scar wound healing and tissue regeneration in human skin samples depend on non-muscle myosin II (NMMII) expression in scar fibroblasts, leading to collagen matrix contraction,^197^ suggesting that CSK prestress is important in regeneration.

Tensegrity has been proposed as a physical basis to explain how molecular elements assemble to create hierarchical structures with increasingly complex functions, including living cells that can reproduce.^198^ We posit that homeostasis of endogenous energy-dependent tensegrity-like, stress-supported structures that use CSK prestress to stabilize structure represents a biological principle that is not observed in inorganic systems, whereas other basic principles (e.g., self-assembly) are required for living systems but are also found in simpler nonliving systems. How nature evolves from utilizing physical and chemical laws to emerging biological principles remains unclear (Fig. 3) and is beyond the scope of this study.

## 7 Concluding remarks

In summary, we propose that CSK prestress homeostasis governs living cell structures and functions, and so it is proposed to be a biological principle. CSK prestress homeostasis, together with Central Dogma and metabolism, as well as cell volume and cell shape homeostasis, may underpin the homeostasis of all cells, including endothelial cells^199^ and living tissues. Due to the existence of numerous intrinsic homeostatic regulation in living cells, we must be aware that cells (and the tissues in the body) often respond to external stimuli (mechanical, chemical, or electrical) by restoring the system to physiological homeostasis. When cells or tissues are exposed to perturbations, they come back to homeostasis after certain periods, unless they are exposed to chronic infection or chronic inflammation, long durations of large abnormal mechanical stimuli (e.g., hypertension), or malignant tumor growth, or unless they undergo the process of aging.

Leveraging the biological principle of CSK prestress homeostasis and other to-be-discovered biological principles could have far-reaching implications in understanding the essence of the cell's life that dictates cellular functions in development, physiology, and diseases, and in designing effective interventions for therapeutics to enhance human health and advance medicine.

## CRediT authorship contribution statement

**Fazlur Rashid:** Writing – review & editing, Writing – original draft, Visualization. **Ning Wang:** Writing – review & editing, Writing – original draft, Visualization, Validation, Supervision, Resources, Project administration, Methodology, Investigation, Funding acquisition, Formal analysis, Conceptualization.

## Ethical approval

This study does not contain any studies with human or animal subjects performed by any of the authors.

## Funding

10.13039/100000002National Institutes of Health grant R01 GM072744 (10.13039/100018068NW) and 10.13039/100015257Northeastern University in Boston (10.13039/100018068NW). FR was supported by the 10.13039/100005302University of Illinois at Urbana-Champaign.

## Declaration of competing interest

The authors declare that they have no known competing financial interests or personal relationships that could have appeared to influence the work reported in this paper.

## Data Availability

All publications cited in this review are in the public domain. There is no original experimental data generated in this work.
